# Molecular Health Effects of Electronic Cigarettes

**DOI:** 10.3390/biom16020264

**Published:** 2026-02-07

**Authors:** Paweł Sutkowy, Igor Hadryś, Wiktor Gmys, Przemysław Grzempa, Aleksandra Sobieszczańska, Weronika Tuska, Karolina Błachnio, Alina Woźniak

**Affiliations:** 1 Department of Medical Biology and Biochemistry, Faculty of Medicine, Ludwik Rydygier Collegium Medicum in Bydgoszcz, Nicolaus Copernicus University in Toruń, 24 Karłowicza St., 85-092 Bydgoszcz, Poland; al1103@cm.umk.pl; 2 Student Research Club of Medical Biology and Biochemistry, Department of Medical Biology and Biochemistry, Faculty of Medicine, Ludwik Rydygier Collegium Medicum in Bydgoszcz, Nicolaus Copernicus University in Toruń, 24 Karłowicza St., 85-092 Bydgoszcz, Poland; 327428@stud.umk.pl (I.H.); wmgmys@gmail.com (W.G.); 325509@stud.umk.pl (P.G.); 326417@stud.umk.pl (A.S.); 330302@stud.umk.pl (W.T.); 330806@stud.umk.pl (K.B.)

**Keywords:** oxidative stress, inflammation, electronic cigarettes

## Abstract

Electronic cigarettes (e-cigarettes) have emerged as a prevalent substitute for conventional cigarettes, garnering perceptions of being a safer option for health. Nicotine addicts use e-cigarettes to cease smoking. These products have also become common among young people because of their taste, smell, and attractive appearance. However, accumulating experimental and clinical evidence indicates that e-cigarette use is not risk-free. The inhalation of e-cigarette aerosols exposes users and their non-using peers to a complex mixture of chemical compounds, including aldehydes, heavy metals, and flavoring agents, many of which possess pro-oxidative and pro-inflammatory properties. This review summarizes and critically analyzes current evidence on the molecular and cellular mechanisms underlying the biological effects of e-cigarette aerosols. Particular attention is given to excessive production of reactive oxygen species, mitochondrial dysfunction, DNA damage, and the activation of redox-sensitive signaling pathways, including NF-κB and NRF2. These molecular alterations may trigger acute and, with prolonged exposure, chronic oxidative stress and inflammation, which in turn can affect gene expression, protein function, and metabolic pathways. While molecular and experimental studies often demonstrate adverse biological responses to e-cigarette aerosols, the translation of these findings into long-term clinical outcomes remains an area of ongoing investigation.

## 1. Introduction

The promotion of alternative smoking methods and nicotine delivery to an organism by tobacco companies has contributed significantly to the popularity of electronic smoking: electronic nicotine delivery systems (ENDS) and electronic non-nicotine delivery systems (ENNDS) [1]. These electronic systems primarily concern electronic cigarettes (e-cigarettes), which are referred to as vapes, and the act of using them is called vaping. E-cigarettes have dominated the market. E-cigarettes can be classified as either ENDS or ENNDS, depending on whether the product contains nicotine [2]. Contrary to common belief, e-cigarette use is not risk-free and may be comparable to traditional cigarette smoking in terms of health risks. In addition, it does not only reduce the effectiveness of quitting smoking but also, at a population level, e-cigarette use may increase the number of cigarette smokers [1,2]. Lung damage and toxic substances, including carcinogens and heavy metals, are just a few examples of the risks associated with e-cigarettes [2]. As early as 2013, the WHO had been reporting on the growing problem of e-cigarette use, alerting that it is already an epidemic and seriously hazardous because it mainly affects young people [1]. In addition, during the recent pandemic, information regarding the protective role of nicotine against COVID-19 was disseminated [3]—the WHO strongly warned against this misleading information [4]. Indeed, e-cigarette use has been demonstrated to augment the probability of contracting SARS-CoV-2, potentially, and it has been associated with an elevated risk of developing a more severe case of COVID-19 in comparison to individuals who do not use e-cigarettes. This is due to inflammation and compromised respiratory and immune functions [3]. A meta-analysis conducted in 2021 has confirmed that e-cigarette use is harmful to the lungs, contributes to asthma and chronic obstructive pulmonary disease (COPD), and causes many biological disorders in the lung tissues, which increase susceptibility to infections of this organ [5]. E-cigarettes have the potential to elicit allergic symptoms in a manner analogous to that of conventional tobacco smoking (asthma, allergic rhinitis, and atopic dermatitis) [6]. E-cigarettes affect the respiratory epithelium in a similar way to traditional cigarette smoking. This concerns impaired host defense responses and immune suppression in the lungs, due to reduced expression of relevant genes [7], including those involved in DNA damage (genotoxicity) [8]. E-cigarette-related toxicity also affects other tissues and involves virtually the entire organism [9,10]. The toxicity observed can be attributed to a number of substances, including heavy metals, volatile organic compounds (e.g., flavorings), and polycyclic hydrocarbons (e.g., benzopyrene) [10,11,12]. The underlying cause of this toxicity is most likely oxidative stress, defined as excessive concentrations of reactive oxygen species (ROS) [13]. Damage to cell components, including DNA, and inflammation are possible consequences [14]. At the organism level, this may result in a wide range of acute and chronic pathological states of tissues and organs, including carcinogenic processes [15,16]. Consequently, this review aims to summarize and critically analyze current experimental and clinical evidence on the molecular and cellular mechanisms underlying the biological effects of e-cigarette aerosols. Particular emphasis is placed on oxidative stress and inflammatory signaling pathways, and on their downstream consequences at the levels of genes, proteins, and metabolic pathways. Furthermore, this review discusses how mechanistic findings derived from in vitro and animal studies may relate to observed organ-level and clinical outcomes, while highlighting existing limitations and gaps in the available evidence.

## 2. Electronic Smoking Methods

ENDS are typically associated with electronic cigarettes, which are devices that deliver nicotine by heating and atomizing a solution of vegetable glycerin (VG) and/or propylene glycol (PG) in combination with nicotine and aromas (e-liquid) [17,18,19]. As mentioned, electronic smoking methods can be employed without delivering nicotine (ENNDS) [1].

**Figure 1:**
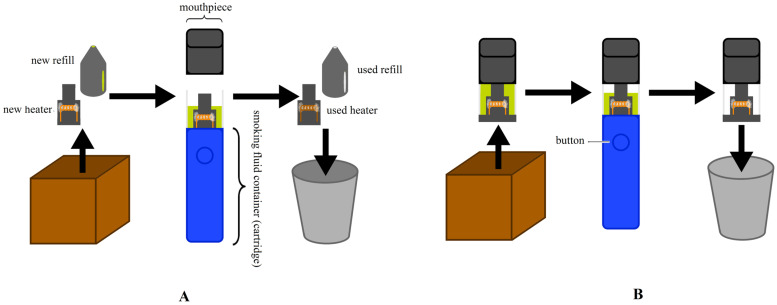
Electronic cigarettes. (**A**): open system; (**B**): closed system.

E-cigarettes are composed of a mouthpiece, a battery, an e-liquid container, and a heater (Figure 1). The latter is a metal wire wrapped around a piece of cotton wool that heats up when electricity flows through it. The cotton wool is continuously saturated with e-liquid from the tank, which is released in the form of an aerosol when it comes into contact with the heated metal [20]. The activation of the mechanism is contingent upon the specific model and manufacturer. In some cases, the mechanism is activated automatically upon the detection of airflow through the mouthpiece. In other cases, the mechanism is activated manually through the pressing of a button. E-cigarettes can be categorized into two distinct classifications: open and closed systems. The adaptability of open systems allows for the addition of e-liquid of diverse flavor profiles and nicotine concentrations, the adjustment of voltage and power settings, and the replacement of heaters and batteries as necessary (Figure 1A). Closed systems refer to devices in which entire cartridges (refills) are replaced, that is to say, e-liquid containers with a built-in heater. Closed systems also include disposable e-cigarettes, which are characterized by the absence of replaceable components (Figure 1B) [17,18,19].

**Figure 2:**
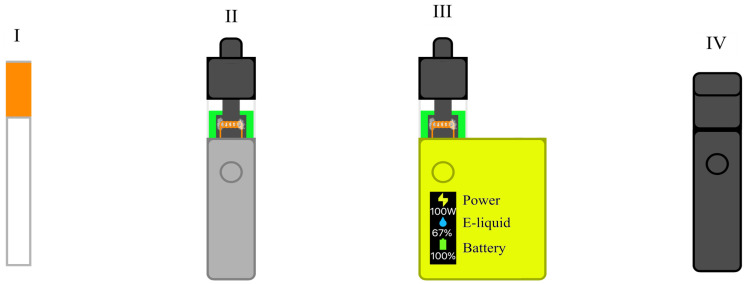
The generations of e-cigarettes. Generation I—e-cigarette-like devices, Generation II—refillable cartridges, Generation III—customizable devices, Generation IV—e-liquid with nicotine salts.

The generations of e-cigarettes mark the technological evolution of these devices. They have evolved from simple, e-cigarette-like devices (Generation 1) to larger, refillable devices with refillable cartridges (Generation 2), then to customizable “mods” with adjustable settings and powerful batteries (Generation 3), and finally to compact “pod systems” with replaceable cartridges that use nicotine salts to deliver a milder sensation while providing a higher nicotine dose (Generation 4) (Figure 2) [1,2,17,18,19].

## 3. Chemical Composition of Electronic Cigarette Aerosols

### 3.1. Heavy Metals

Typical e-liquid compositions include PG, VG, or a mixture of the two, as well as flavorings that provide the e-cigarette with its flavor profile. The potential harmful effects of electronic cigarettes are largely contingent upon the composition of the e-liquids utilized. During heating in the device, these liquids evaporate, generating an aerosol that causes the associated harmful effects. Nicotine is an optional additive [21]. Despite the simplicity of e-liquid composition, the substances inhaled by the user may differ significantly from those originally found in the e-liquid. This phenomenon is attributable to the processes occurring during the aerosol production, which can result in the release of various metals from the e-cigarette’s heating elements [22]. Manufacturers employ a variety of metals and production methods in the manufacture of these components, resulting in a composition of metals in the aerosol that varies between products of the same brand. The most prevalent metals include: nickel (Ni), lead (Pb), chromium (Cr), copper (Cu), cadmium (Cd), manganese (Mn), and tin (Sn) [16,22,23,24,25,26,27,28,29,30]. The first four of these have been observed in electronic cigarette refill liquids, which may be attributed to contamination during the manufacturing process [31]. Arsenic (As) or zinc (Zn) have also been detected in aerosols generated by electronic cigarettes, although their presence is observed less frequently [16,22,24,25,26,27,28,30] (Table 1).

### 3.2. Volatile Organic Compounds

The use of e-cigarettes involves the thermal decomposition of e-liquid organic components (PG, VG), resulting in the formation of novel compounds, including volatile organic compounds (VOCs). The most common VOCs found in e-cigarette aerosol are various types of carbonyl compounds, including aldehydes such as formaldehyde, acetaldehyde, and acrolein [16,23,32,33,34,35,36,37,38,39,40,41,42]. VOCs also encompass flavorings and their derivatives [35]. The VOC flavorings that can be detected in e-cigarette aerosols are: acetol, 3-hexen-1-ol, diacetyl, methyl propionate [35], menthol, vanillin, cinnamaldehyde, benzyl alcohol, benzaldehyde [43,44], glyoxal, methylglyoxal [38,41] (Table 1).

### 3.3. Polycyclic Aromatic Hydrocarbons

Additionally, polycyclic aromatic hydrocarbons (PAHs) have also been detected in e-cigarette aerosol: naphthalene, chrysene [38], benz[a]anthracene, benzo[b]fluoranthene, benzo[k]fluoranthene, benzo[a]pyrene, dibenz[a,h]anthracene, benzo[g,h,i]perylene [45]. The listed substances have been classified in International Agency for Research on Cancer (IARC) group 2B, with the exception of benzo[a]pyrene, dibenz[a,h]anthracene, and benzo[g,h,i]perylene, which have been classified in groups 1, 2A, and 3, respectively [46]. The assignment of a substance to group 1 indicates a determination of its carcinogenicity to humans. Substances classified in group 2A are likely carcinogenic to humans, while those in group 2B are considered possibly carcinogenic. The assignment of the substance to group 3 indicates that it could not be classified in other groups due to an absence of sufficient evidence of carcinogenicity in humans and experimental animals [46] (Table 1).

**Table 1:** A list of the substances found in electronic cigarette aerosols, accompanied by their Chemical Abstracts Service (CAS) number and classification according to the International Agency for Research on Cancer (IARC) [46].

Substance	CAS Registry	IARC
Heavy metals:		
Nickel	7440-02-0	2B
Lead	7439-92-1	2B
Chromium	7440-47-3	3
Copper	-	-
Cadmium	7440-43-9	1
Manganese	-	-
Tin	-	-
Arsenic	7440-38-2	1
Zinc	-	-
*VOCs—aldehydes:*		
Formaldehyde	50-00-0	1
Acetaldehyde	75-07-0	2B
Acrolein	107-02-8	2A
Cinnamaldehyde	-	-
Benzaldehyde	-	
Other VOCs:		
Propylene glycol	-	
Vegetable glycerin	-	
Acetol	-	
3-hexen-1-ol	-	
Diacetyl	-	
Methyl propionate	-	
Menthol	-	
Vanillin	-	
Benzyl alcohol	-	
Glyoxal	-	
Methylglyoxal	3	
PAHs:		
Naphthalene	2B	
Chrysene	2B	
Benz[a]anthracene	2B	
Benzo[b]fluoranthene	2B	
Benzo[k]fluoranthene	2B	
Benzo[a]pyrene	1	
Dibenz[a,h]anthracene	2A	
Benzo[g,h,i]perylene	3	

VOCs: volatile organic compounds; PAHs: polycyclic aromatic hydrocarbons.

## 4. Molecular and Cellular Effects of E-Cigarette Use

### 4.1. Oxidative Stress, Inflammation and Associated Cellular Alterations—In Vitro Studies

Numerous studies have described the molecular mechanisms underlying the toxic effects of e-cigarettes on cells. For instance, e-cigarette samples substantially increased ROS concentration and inflammatory interleukin production (IL-6, IL-8, and IL-1β) regardless of nicotine, ultimately leading to cell death in human models of monocytes/macrophages (THP-1, MM6, and U937 cells) and airway epithelial cells (BEAS-2B and H292 cells) [47,48,49]. Similar findings were obtained by Hwang et al., who observed that exposure of human epithelial cells (keratinocytes, CLS, and lung alveolar type II epithelial cells, A549) to e-cigarette aerosol from an e-cigarette device resulted in dose-dependent cell death [50]. Moreover, after exposure to the e-cigarette aerosol, epithelial cells, alveolar macrophages, and neutrophils had reduced antimicrobial activity against Staphylococcus aureus [50]. In turn, in human lung fibroblasts (HFL-1), electronic cigarette aerosols and copper nanoparticle-containing aerosols induced mitochondrial stress (ROS production) and promoted DNA fragmentation. The authors of the study evaporated a copper solution to imitate copper ions that may be present in e-cigarette aerosols [51]. Heavy metals were found in e-cigarette users in saliva samples [52], urine [52,53,54], blood/serum [54,55], and exhaled air [52]. However, some research has reported no evidence of heavy metals in the bodies of e-cigarette users. Blood Pb levels and urinary Cd, Ba, and Sb levels were similar between e-cigarette users and non-users [56]. Heavy metal ions, such as those belonging to the transition metal family (e.g., Hg, As, Cr, Ni, Se, Co, Mn, Mo, Cu, and especially Fe), can interfere with the redox balance in biological systems. These ions are considered an important source of ROS, which generate the highly reactive hydroxyl radical (˙OH) via the Fenton/Fenton-like reaction when hydrogen peroxide (H_2_O_2_) breaks down [57]. Ultimately, this usually entails the production of other ROS and oxidative stress [58]. Conversely, Cd has been demonstrated to induce oxidative stress by inhibiting antioxidant enzymes, including catalase, superoxide dismutase, and glutathione peroxidase [59]. For instance, data from BEAS-2B cell models have shown increased ROS production following exposure to Cr [60] and Ni [61]. Nickel triggered ROS-mediated apoptosis by activating the c-Myc gene via the ERK pathway [61]. Cd exhibited consistent action, resulting, however, in cellular proliferation. The observed resistance to apoptosis was attributed to mutations in the *BCL-2*, *p53*, and *BAX* genes, which may contribute to the transformation of epithelial cells into cancer cells [62,63]. Furthermore, other heavy metals, such as Pb, can cross-link proteins and DNA. Pb can also bind to DNA phosphate groups, altering its shape. These changes may result in chromosomal aberrations and gene mutations [64].

Another study examined the newest, fourth generation of e-cigarettes, characterized by disposable cartridges and nicotine salts for more effective nicotine delivery. The study reports that the e-cigarette aerosol without nicotine significantly decreased BEAS-2B cell viability, and the aerosol containing nicotine was even more toxic and associated with a genotoxic effect (DNA damage) (24-h exposure) [65]. However, that study observed a slight adverse effect in a human lung carcinoma cell line derived from an adenocarcinoma (A549 cells, a model for studying lung cancer) and in human distal lung tissue explants [65]. The effect of e-cigarettes on ROS concentration may have consequences in cancer treatment. It has been demonstrated that squamous cell carcinoma cells treated with cisplatin and exposed to e-cigarette aerosol showed increased survival, and the concentration of cisplatin required to inhibit cell growth by 50% was significantly higher than that in cells not exposed to the aerosol [66].

Some studies suggest that e-cigarettes may be safer than traditional cigarettes. For instance, Tylor et al. found that extracts of commercial e-cigarettes (Generation 1 and 2) did not inhibit migration of human umbilical vein endothelial cells (HUVECs) following artificial wounding, allowing cells to migrate into the wounded area. Those extracts did not stop migration, even at a nicotine concentration two times higher than that used in the extract of a scientific reference cigarette. In turn, exposure of the cigarette extract induced concentration-dependent inhibition of endothelial cell migration, with complete inhibition at concentrations above 20% [67]. However, Anderson et al. found that exposure to tobacco-flavored, cigarette-like e-cigarettes (Generation 1) causes HUVECs to generate significantly more ROS, leading to DNA damage and cell death through necrosis (phosphorylated mixed lineage kinase domain-like detection) or apoptosis (active caspase-3 detection) [68]. The study demonstrated that this cytotoxicity was dose-dependent and that the antioxidant α-tocopherol partially counteracted the cell death, confirming the causal role of ROS in toxic effects [68]. Furthermore, blood plasma from young healthy nicotine e-cigarette users added to the culture medium of endothelial cells (EA. Hy 926) and epithelial cells (A549) increased mitochondrial membrane potential and ROS levels. It decreased maximum mitochondrial respiration and cell reserve capacity (metabolic stress). Electron microscopy revealed structural changes in the mitochondria [69]. E-cigarettes, especially flavored ones, primarily cause a dose-dependent loss of pulmonary endothelial barrier function [70,71,72]. The disruptive effects may be associated with increased intracellular ceramides, p38 MAPK activation, and myosin light chain (MLC) phosphorylation. These effects are mediated by Rho-activated kinase via inhibition of the MLC phosphatase, MYPT1. The result is inflammation and loosening of cell connections (increased endothelial permeability). These effects could be attributed to acrolein, which was detected alongside PG and glycerol in both e-liquid and the aerosol [70]. Some authors reported that aerosol produced from pure PG may be more toxic than that produced from pure VG, and e-cigarette toxicity results in ROS and aldehydes produced during e-liquid evaporation in the device [47]. It is suspected that mainly aldehydes of the VOC type are responsible for the harmful effects of e-cigarette use on the human body, especially formaldehyde, acetaldehyde [47,73,74,75], and acrolein [70,74,75]. They are produced during the heating of PG and/or VG [47,76] and have carcinogenic properties [46]. Although e-cigarettes emit fewer types of VOCs than traditional cigarettes, VOC concentrations are higher during e-cigarette use [34]. Studies have shown that the aldehydes, in conjunction with ROS, can contribute to DNA damage. Formaldehyde can lead to alkylative and oxidative DNA lesions, accompanied by increased ROS concentrations [73]. Chronic exposure to e-cigarette aerosol increased the expression of excision repair cross-complementation group 1 (ERCC1) protein in human premalignant dysplastic oral mucosal keratinocytes (POE9n). ERCC1 protein is essential for nucleotide excision repair. In contrast, the exposure contributed to a statistically significant decrease in the concentration of 8-oxoguanine-DNA glycosylase, a protein involved in base excision repair, in POE9n and human oral squamous cell carcinoma (UM-SCC-1) cells [73]. Aldehyde interaction is also associated with the formation of irreversible, unreducible form of glutathione (aldehyde-GSH adducts) [77].

Acrolein [78] is probably the most destructive substance responsible for the non-nicotine-related consequences of e-cigarette use on the body. It has been detected using nuclear magnetic resonance, mass spectrometry, and gas chromatography, not only in the aerosols but also in refill liquids [70]. It is an aldehyde with electrophilic properties that binds to nucleophilic side-chain residues of proteins and DNA [79]. It is also suggested that it may activate the NADPH oxidase (NOX) complex by binding to its thiol groups [80]. Acrolein likely affects endothelial intercellular tethering molecules [70] and can directly or indirectly (glutathione oxidation) activate nuclear factor erythroid 2-related factor 2, NRF2 [81]. Thus, acrolein can lead to excessive ROS concentrations and may increase endothelial permeability. NRF2 activation is a typical cellular defense response to oxidative stress and increased concentrations of electrophilic substances, as it results in the transcription of many antioxidant defense genes, including antioxidant enzymes [82], as well as pro-inflammatory genes, as NRF2 activation is combined with, among others, nicotine-independent nuclear factor kappa B (NF-κB) pathway activation [83]. A study by Kuntic et al. conducted on endothelial (EA.hy 926) and macrophage (RAW 264.7) cell lines showed that acrolein contributes to a dose- and time-dependent reduction in the survival of both cell types [78]. This was associated with increased ROS production by the cells. In EA.hy 926 cells, this production was mainly intracellular, whereas in RAW 264.7 cells, it was extracellular. An increase in Rac1 protein concentration in the cell membrane was also observed in both lines, indicating NOX complex activation [78].

Others indicated flavor-aroma compounds as a causative agent [48,49], and even unevaporized e-liquids without nicotine may have oxidative properties (ROS) [49]. Sweet or fruit flavors seem to be more potent oxidizers than tobacco flavors, and mixing of flavors is more harmful to the users than single-flavor use [49]. Flavor compounds utilized in e-cigarettes have the potential to induce many physiological disorders. This is particularly true for e-cigarettes, which offer a diverse range of flavor options [1,2]. The human cell line HEK293T treated with e-liquids exhibited a decrease in mitochondrial membrane potential and cellular membrane potential, as well as an increase in ROS production and cellular toxicity in a dose-dependent manner. This was linked with vanillin, benzyl alcohol, acetoin, cinnamaldehyde, and methyl-cyclopentenolone. Significantly, it was not related to nicotine [13]. Furthermore, acute exposure to flavored e-liquids with various nicotine concentrations or e-cigarette use was demonstrated to exacerbate endothelial dysfunction [71]. This finding was revealed using human-induced pluripotent stem cell-derived endothelial cells (iPSC-ECs) exposed to e-liquids and in serum derived from e-cigarette users. The authors observed a decline in cell viability, as evidenced by impaired tube formation and cell migration. Furthermore, increased ROS concentration, caspase 3 and 7 activities, and low-density lipoprotein uptake were also found. The aforementioned effects were particularly associated with cinnamon aroma [71]. Similar outcomes have been demonstrated in human airway epithelial cells (H292) when cinnamon flavor was used [49]. Moreover, treatment of freshly isolated endothelial cells from non-e-cigarette users with either menthol or eugenol (0.01 mmol/L) decreased nitric oxide (NO˙) production [72]. In contrast, human aortic endothelial cells exhibited elevated levels of ROS production, IL-6 expression, and NO˙ production following a 90-min incubation with various flavorings, including vanillin, menthol, cinnamaldehyde, eugenol, dimethylpyrazine, diacetyl, isoamyl acetate, eucalyptol, and acetylpyrazine, with concentrations ranging from 0.1 to 100 mmol/L. The highest concentrations of these flavorings resulted in cell death [72].

### 4.2. Organ-Level Alterations Observed in Mouse Models

In mouse lungs, e-cigarette samples increased pro-inflammatory cytokine production and decreased glutathione concentration [49,50]. The authors found that the samples induced neutrophil infiltration, IL-1β production, heme oxygenase-1 expression, and NF-κB expression, which was associated with acute lung inflammation in mice [47]. NF-κB is one of the most important pathways counteracting oxidative stress. It is composed of five related transcription factors: p50, p52, RelA (p65), c-Rel, and RelB. The NF-κB pathway increases the expression of antioxidant proteins, especially antioxidant enzymes such as superoxide dismutase, catalase, and glutathione peroxidase [84,85]. Moreover, as ROS concentration increases, NF-κB induces the expression of various pro-inflammatory genes, including those encoding cytokines and chemokines, and participates in inflammasome regulation [86]. ROS induce inhibitor kappa B (IκB) kinase activation, leading to the phosphorylation and subsequent degradation of IκBα, thereby enabling NF-κB to translocate to the nucleus and initiate transcription of target genes [87]. Another interesting study, based on immunohistochemical staining of alveolar macrophages from the lungs of male Wistar rats exposed to nicotine e-cigarette aerosols for an extended period (two minutes a day for four weeks), showed an increased number of macrophages expressing malondialdehyde (a marker of oxidative stress), IL-8, IL-10, and matrix metalloproteinase-8, as well as a decreased number of macrophages expressing type II collagen. The authors concluded that lung inflammation was shown, which could be associated with lung microdamage [88]. Cell damage leads to necrosis, resulting in cellular debris in the microenvironment [89]. Cellular debris in the lungs is absorbed by M1 alveolar macrophages [90]. These macrophages are stimulated in this manner to secrete various cytokines that activate the immune response [90]. M1 macrophages promote an inflammatory reaction and secrete, among others, IL-8 [90]. Macrophages also regulate the inflammatory response by secreting anti-inflammatory cytokines, such as IL-10, during excessive inflammation (M2 macrophages) [91]. Moreover, alterations in the expression of immunomodulatory cytokines in the mice’s airways, resulting from inhaling e-cigarette aerosol, led to increased susceptibility to infection by Staphylococcus aureus [50], and the bacteria became more virulent when exposed to the aerosol [50]. Another study confirms these findings, indicating that exposure to e-cigarette aerosol weakens the mouse lung’s immune barrier against both bacteria and viruses [92]. Other data suggest that inhaling e-cigarette aerosol exacerbates allergy-induced asthma symptoms in murine models [93].

A key mechanism by which e-cigarettes may impair lungs and other organs may depend on the phagocytic NOX (NOX-2) in endothelium, an enzyme crucial for immune cells in killing pathogens by producing a ROS cascade from superoxide anion radicals (O_2_^−^) [94]. It has been suggested that even a short-term exposure to e-cigarette aerosol (Generation 4) can markedly disrupt glucose metabolism and augment neuroinflammation [95]. Reduced expression of essential tight junction proteins at the blood–brain barrier (BBB) may be implicated in this process [95]. Nicotine, solvents (PG, VG), and flavors from e-cigarettes may also affect fetus [96]. Studies suggest that prenatal e-cigarette aerosol exposure may increase the risk of future development of respiratory diseases in offspring, such as asthma and COPD [96]. Archie et al. investigated the effects of e-cigarette use on BBB integrity and behavioral outcomes in the offspring of pregnant CD1 mice [97]. Their findings revealed reduced expression of tight junction proteins, such as zonula occludens-1 and occludin, alongside neuroinflammation and behavioral deficits in offspring [97]. In a comparable study, Heldt et al. found that e-cigarette aerosol increased BBB permeability via inflammation, leading to microglial activation in male C57BL/6 mice, which contributed to cognitive impairment [98]. In other study, an increased risk of thrombogenic events was observed in mice [99]. Furthermore, it was observed that acrolein was responsible for the NOX-2-dependent effects of e-cigarette aerosol (oxidative stress, inflammation) on blood vessels (in vitro incubation) in mice lacking NOX-2 or upon treatment with the endothelin receptor blocker macitentan or the FOXO3 activator bepridil [94].

## 5. Clinical and Epidemiological Evidence of Health Effects Associated with E-Cigarette Use

### 5.1. Respiratory System Effects

Inhalation of e-cigarette aerosols poses a higher risk of health complications than exposure through the skin or oral consumption (e-liquids) [16]. The lungs are most vulnerable to the consequences of e-cigarette use [100]. Zhao et al. estimated the risk of non-carcinogenic changes associated with e-cigarettes, and in most cases, the risk was within acceptable limits. However, the assessment of carcinogenic changes showed that e-cigarette use caused an additional 535 cases of cancer per 1,000,000 individuals [16]. A similar finding was reported by Lu et al. Only the non-cancer health risk of e-cigarettes was acceptable (0.91, limit is 1) [33]. In a control case study, traditional cigarette smoking combined with e-cigarette use was associated with a fourfold increase in the risk of lung cancer compared to smoking tobacco alone [101]. There is also a risk of malignant tumors in other organs of respiratory tract, including the mouth, throat, and larynx [102], especially in the case of e-cigarettes with nicotine [103], which is not always strictly controlled (differences between the actual and declared nicotine content in the product) [104]. However, the intensity of e-liquid production may be more important than nicotine content. For example, significant differences in formaldehyde and acetaldehyde concentrations in e-cigarette aerosol were observed depending on the aerosol intensity production [16]. The risk of lung and nasopharyngeal cancer at a constant device power (unregulated e-cigarette use) was more than twice as high for e-liquid containing 6 mg/mL of nicotine than for e-liquid containing 18 mg/mL of nicotine. During regulated e-cigarette use, the risk was also higher at lower nicotine concentration (a difference of 27%) [104]. In general, e-cigarettes may be less harmful than traditional cigarettes, especially when aerosol production is lower [33].

The attractive appearance of e-cigarettes and their variety of flavors encourage their use, create a smoking habit, and lead to nicotine addiction. There has been a rapid increase in the number of e-cigarette users, especially among young people, which sometimes leads to serious consequences [1,2]. Life-threatening bronchiolitis related to electronic cigarette use in a certain 17-year-old male was described, for example [105]. Another study evaluated the risk of bronchitis symptoms. The risk was almost twice as high among past users (OR = 1.85) compared to never-users of e-cigarettes and 2.02 times higher among current users. The risk increased with greater frequency of use (OR = 1.66) [106]. In addition, recent epidemiological studies have shown an association between e-cigarette use and asthma in children [107,108,109] and in adults with respiratory disorders [110]. Adult e-cigarette users who had never smoked conventional cigarettes were more likely to report an asthma diagnosis than those who did not use e-cigarettes [111]. Studies indicate a significant link between e-cigarette use and an increased risk of asthma (aOR = 1.39) and COPD (aOR = 1.49). Moreover, exposure to e-cigarette aerosol has been linked to other respiratory tract pathologies. For example, an e-cigarette user developed giant cell interstitial pneumonia, a rare disease primarily associated with occupational exposure to heavy metals, including cobalt. Analysis of the e-liquid revealed high levels of cobalt, which supports the e-cigarette’s role as the cause of the disease [112].

### 5.2. Cardiovascular and Metabolic Effects

Some studies suggest that e-cigarettes are harmless to vascular function when used without nicotine. Neither sham nor nicotine-free e-cigarette use resulted in modifications of cardiovascular parameters, unlike e-cigarette use with nicotine [113]. However, this study was conducted in habitual tobacco smokers (*n* = 25) [113]. In contrast, a study of healthy non-users indicated dysfunction of the pulmonary blood vessels after a single acute nicotine-free e-cigarette session (disposable, tobacco-flavored e-cigarettes filled with 70% PG and 30% VG; *n* = 10 and *n* = 31) [114,115]. A cross-sectional study of habitual e-cigarette users (*n* = 42) found higher concentrations of oxidized low-density lipoproteins and a shift in cardiac autonomic balance toward sympathetic predominance [116]. However, there is no doubt that e-cigarettes containing nicotine may be as harmful as cigarettes. An analysis of 9 clinical studies involving 370 people showed that short-term nicotine e-cigarette use results in vascular endothelial dysfunction comparable to that caused by smoking regular cigarettes [117]. Additionally, changes in exosome profiles were found in the blood plasma of young, healthy nicotine e-cigarette users, with significantly increased concentrations of free mitochondrial DNA, carbonyl proteins, and 4-hydroxynonenal (the latter two are oxidative stress markers) [69].

A population study of 460,603 Korean adults considered variables such as age, gender, education level, region, occupation, alcohol consumption, sports activity, health status, body weight, smoking of cigarettes or e-cigarette use, and the prevalence of diabetes, which was reported by 12.9% of respondents [118]. It was shown that e-cigarette users had a 15% higher risk of developing diabetes and a high risk of dyslipidemia compared to non-users. This risk was higher for traditional cigarettes, at 22%. People who use both electronic and traditional cigarettes are at the highest risk of developing diabetes, at 39% [118]. Furthermore, a US study of 5101 people found that e-cigarette use is associated with increased insulin resistance (OR = 1.22) [119].

### 5.3. Second-Hand Exposure and Vulnerable Populations

The final issue concerns second-hand exposure to e-cigarettes, particularly in vulnerable populations such as pregnant women and children. Direct exposure studies involving humans indicated that passive exposure to e-cigarette aerosol is associated with nicotine uptake from the air similar to that from conventional cigarettes, even when the air nicotine concentration is higher with cigarettes. However, the studies did not reveal any inflammatory changes in the body (saliva, urine, serum) with passive inhalation of e-cigarette aerosols, in contrast to the effects observed with passive inhalation of tobacco smoke [120]. Furthermore, there is also research that shows no impact of e-cigarettes on asthma symptoms in children. For example, a pilot, retrospective, monocenter, observational study (N = 57, individuals aged 5–17 years), based on demographic and clinical data, demonstrated that the number of asthma symptomatic days and need for a therapeutic step-up were similar in asthma patients exposed to e-cigarette aerosols at home as compared to similar group without exposition to e-cigarette aerosols (controls) [121].

Due to a limited number of studies with small sample sizes, this issue remains under debate and requires further research.

## 6. Conclusions

The growing diversity of e-cigarette products, including a wide range of flavorings and device designs, has contributed to their increasing use, particularly among adolescents and young adults. Although e-cigarettes are often perceived as less harmful than conventional cigarettes, accumulating experimental evidence indicates that exposure to e-cigarette aerosols can perturb fundamental cellular homeostasis.

Across multiple in vitro and animal models, a recurrent and central biological mechanism associated with e-cigarette aerosol exposure is redox imbalance, characterized by excessive ROS production. This oxidative burden may induce acute and, with prolonged exposure, chronic oxidative stress, leading to activation of redox-sensitive signaling pathways and subsequent inflammatory responses. Notably, the magnitude and nature of these effects appear to depend on aerosol composition, device characteristics, and exposure conditions.

At the molecular level, oxidative stress contributes to mitochondrial dysfunction, DNA damage, and altered regulation of cell-survival pathways, including NF-κB and NRF2. Activation of NF-κB is primarily associated with pro-inflammatory signaling, whereas NRF2 functions predominantly as a protective and adaptive transcriptional regulator of antioxidant and cytoprotective responses. While NRF2 activation represents an important defense mechanism, sustained oxidative stress may overwhelm these protective pathways, thereby promoting persistent inflammation and cellular injury.

Experimental findings further suggest that molecular and cellular disturbances induced by e-cigarette aerosols may translate into functional impairments at the tissue and organ levels, including disruption of epithelial and endothelial barriers, pulmonary inflammation, vascular dysfunction, and neuroinflammatory changes. However, it is important to emphasize that these observations are primarily derived from experimental models, and their direct relevance to long-term human health outcomes requires careful interpretation.

Evidence regarding carcinogenic potential indicates that e-cigarette aerosols contain compounds with genotoxic and carcinogenic properties, and experimental studies demonstrate DNA damage and impaired DNA repair mechanisms. Nevertheless, the extent to which these molecular alterations translate into clinically relevant cancer risk in humans remains an area of ongoing investigation, particularly given the limited duration of epidemiological follow-up studies.

Overall, current evidence suggests that e-cigarette use is not biologically inert and may elicit adverse molecular and cellular responses under certain exposure conditions. These findings underscore the need for continued mechanistic research and well-designed clinical and epidemiological studies to define exposure–risk relationships better, identify susceptible populations, and clarify the long-term health implications of e-cigarette use and second-hand aerosol exposure.

## Data Availability

Not applicable.

## Abbreviations

ENDS	electronic nicotine delivery systems
ENNDS	electronic non-nicotine delivery systems
E-cigarettes	electronic cigarettes
ROS	reactive oxygen species
VG	vegetable glycerin
PG	propylene glycol
VOCs	volatile organic compounds
PAHs	polycyclic aromatic hydrocarbons
